# A novel approach for the biosynthesis of silver nanoparticles using the defensive gland extracts of the beetle, *Luprops tristis* Fabricius

**DOI:** 10.1038/s41598-023-37175-0

**Published:** 2023-06-22

**Authors:** Anthyalam Parambil Ajaykumar, Ovungal Sabira, Merin Sebastian, Sudhir Rama Varma, Kanakkassery Balan Roy, Valiyaparambil Sivadasan Binitha, Vazhanthodi Abdul Rasheed, Kodangattil Narayanan Jayaraj, Attuvalappil Ravidas Vignesh

**Affiliations:** 1 Division of Bio-Nanomaterial, Department of Zoology, Sree Neelakanta Government Sanskrit College, Pattambi, Kerala India; 2 Clinical Sciences Department, Centre for Medical and Bio-Allied Health Sciences Research, Ajman University, Ajman, United Arab Emirates; 3 Department of Chemistry, Sree Neelakanta Government Sanskrit College, Pattambi, Kerala India; 4 Department of Zoology, Sree Narayana College, Nattika, Thrissur, Kerala India; 5 Basic Sciences Department, Centre for Medical and Bio-Allied Health Sciences Research, Ajman University, Ajman, United Arab Emirates

**Keywords:** Biotechnology, Materials science, Nanoscience and technology

## Abstract

Discovering novel natural resources for the biological synthesis of metal nanoparticles is one of the two key challenges facing by the field of nanoparticle synthesis. The second challenge is a lack of information on the chemical components needed for the biological synthesis and the chemical mechanism involved in the metal nanoparticles synthesis. In the current study, microwave-assisted silver nanoparticle (AgNP) synthesis employing the defensive gland extract of Mupli beetle, *Luprops tristis* Fabricius (Order: Coleoptera; Family: Tenebrionidae), addresses these two challenges. This study was conducted without killing the experimental insect. Earlier studies in our laboratory showed the presence of the phenolic compounds, 2,3-dimethyl-1,4-benzoquinone, 1,3-dihydroxy-2-methylbenzene, and 2,5-dimethylhydroquinone in the defensive gland extract of *L. tristis*. The results of the current study show that the phenolic compounds in the defensive gland extract of the beetle has the ability to reduce silver ions into AgNPs and also acts as a good capping and stabilizing agent. A possible mechanism for the reduction of silver nitrate (AgNO_3_) into AgNPs is suggested. The synthesized AgNPs were characterized by Ultraviolet–Visible (UV–Vis) spectroscopy, Fourier-transform infrared spectroscopy (FTIR), scanning electron microscopy energy-dispersive X-ray (SEM–EDX) analysis and high-resolution transmission electron microscopic (HR-TEM) techniques. The stability of biologically synthesized nanoparticles was studied by zeta potential analysis. The TEM analysis confirmed that AgNPs are well dispersed and almost round shaped. The average size of nanoparticle ranges from 10 to 20 nm. EDX analysis showed that silver is the prominent metal present in the nanomaterial solution. The AgNPs synthesized have antibacterial property against both *Staphylococcus aureus* and *Escherichia coli*. Radical scavenging (DPPH) assay was used to determine the antioxidant activity of the AgNPs. AgNPs exhibited anticancer activity in a cytotoxicity experiment against Dalton’s lymphoma ascites (DLA) cell line.

## Introduction

The experimental organism, *Luprops tristis* Fabricius (Order: Coleoptera; Family: Tenebrionidae), is a detritus-feeding beetle whose distribution stretches from tropical Africa to Asia and the East Indies to Papua New Guinea^1^. Large invasions of the litter-dwelling beetle *L. tristis*, numbering from 0.5 to over 4 million per residential buildings after summer showers, and their lengthy state of dormancy are a common occurrence in rubber plantation regions along the western slopes of the southern region of the Western Ghats, especially Kerala and Tamil Nadu states of India^2^. The adult beetle possesses two tiny defensive glands that measure about 0.8–0.9 mm in size. When agitated, the beetles secrete an odoriferous substance from their defensive gland that causes blisters on human skin. The defensive glands are invaginations of the intersegmental membrane between the seventh and eighth sternites, which expand backward and evert when the abdomen is pressed^3^. When the beetle is agitated this gland is ruptured by rubbing with the hind tarsus for the release of the secretion as a part of a defense mechanism against the enemies. Recent studies in our laboratory showed the presence of biomolecules such as 2,3-dimethyl-1,4-benzoquinone, 1,3-dihydroxy-2-methylbenzene, 2,5-dimethylhydroquinone, tetracosane, oleic acid, hexacosane, pentacosane, 7-hexadecenal and tert-hexadecanethiol in the defensive extract of *L. tristis*^4^.

Nanotechnology is one of today’s most significant scientific advancements. It relies on the production and manipulation of nanoparticles, which necessitates substantial alterations to the characteristics of metals^5^. Nanomaterials have structures with at least one or two dimensions in the 1–100 nm range^6–8^. Nanoparticles act as a link between bulk materials and atomic or molecular structures. The size-related characteristics of nanoparticles can be considerably different from those of bulk materials or fine particles. It is believed that the unique and fascinating features of nanoparticles are due to their large surface area relative to bulk materials. The most difficult and inspiring fields of nanoscience are those involving the synthesis of metal nanoparticles. Metal nanoparticles synthesized by biological route exhibit a range of potential applications in a variety of fields, including healthcare^6,9,10^, agriculture^11,12^, environmental studies, robotics, energy, information technology, aeronautics, mass communication, heavy industry, consumer goods^13^, and the development of different sensors^14–17^. Additionally, these environmentally friendly metal nanoparticles are used in a variety of diagnostic agents, therapeutic drug, and gene delivery, and in treatments for a number of infectious and non-infectious diseases, as well as neurodegenerative and cardiovascular conditions.Silver based chemical compounds have been used for centuries as an antimicrobial^18^ to fight infections and prevent spoilage^19^. Currently, the FDA has allowed the use of silver nitrate and silver nanoparticles (AgNPs) in antibacterial surface impregnation, food supplements, and medical devices^20^. According to earlier research biosynthesized AgNPs using *Jatropa curcas* seed cake extract showed significant antibacterial properties^21^. Green synthesized bimetallic Au–Ag core–shell nanoparticle using *Ocimum tenuiflorum* leaf extract showed antimicrobial properties against Gram+ and Gram−ve bacteria^22^. Additionally, AgNPs exhibit their anticancer action; AgNPs have so far been shown to have significant anticancer properties against cancer cell lines such as MCF-7 breast cancer cells^23–25^, HCT116 colon cancer cells^26^, prostate cancer cells^27^, HeLa cells^28^, lung carcinoma A549 cells^29^, etc.

The scientific community has spent a significant amount of energy and time establishing appropriate synthetic methods for fabricating nanoparticles because of the physiochemical characteristics and numerous applications of nanoparticles. However, the environmental pollution caused by heavy metals restricts the utilization of several physiochemical methods for the synthesis of metal nanoparticles. The biological synthesis of nanoparticles has emerged as a new trend in nanoparticle synthesis due to its nontoxicity, repeatability in manufacturing, ease of scaling up, and well-defined morphology.Phenolic acids serve as both stabilisers and reducing agents in the synthesis of metal nanoparticles^30,31^. The use of phenolic acids is a reliable, simple, efficient, and cost-effective method for producing metal nanoparticles. Since the defensive gland extract of the beetle *L. tristis* is a rich source of phenolic compounds^4^, the current attempt has the main objective to synthesize AgNPs using these compounds. Identification of novel natural resources for the biological synthesis of metal nanoparticles is one of the two key challenges faced by the field of metal nanoparticle synthesis. The second challenge is a lack of information on the chemical components needed for biological synthesis and the chemical mechanism involved in the metal nanoparticle synthesis. In the current investigation, microwave-assisted AgNPs synthesis employing the defensive gland extract from the Mupli beetle, *L. tristis*, addresses these two problems. Another noteworthy fact is that, the experiment insect, *L. tristis* was not killed in the process of extracting the defensive gland. Biologically synthesized AgNPs were tested for antimicrobial, cytotoxicity, and radical scavenging assays.

## Methodology

### The experimental insect

**Figure 1:**
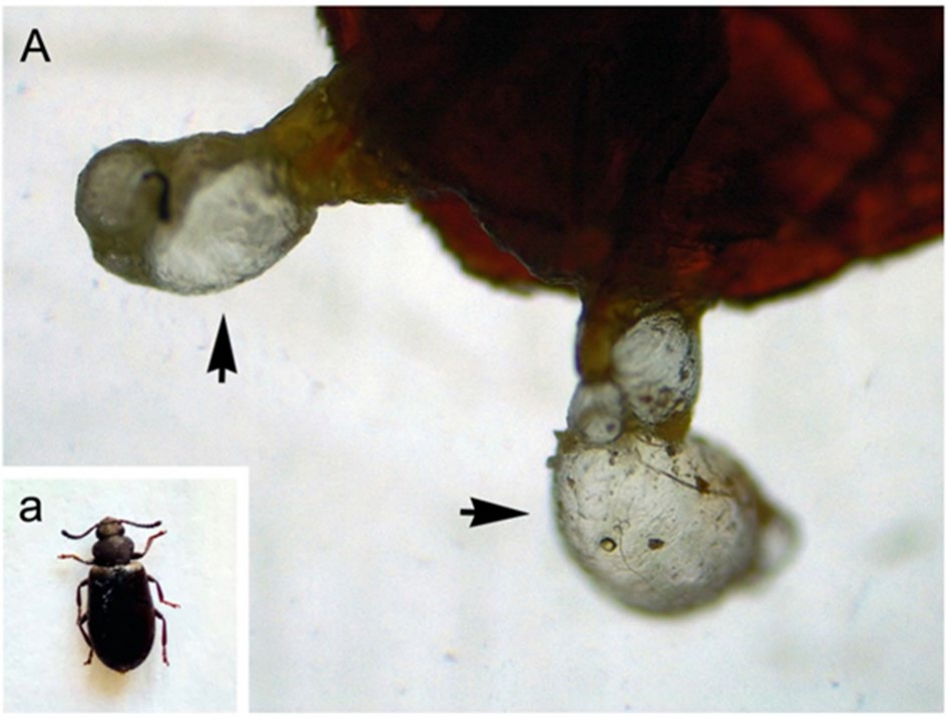
The experimental insect, *L. tristis* (**a**), with exposed defensive gland (**A**).

The experimental insect, *L. tristis* (Order: Coleoptera; Family: Tenebrionidae), is a plant-detritus-feeding darkling beetle found in various parts of India. The adult beetle is black and around 8 mm long. They are usually harmless to humans. When squeezed or picked up, they produce a defensive phenolic secretion that causes skin burns. They have a notorious reputation as they can make life difficult when large populations invade farm houses, roofs of hoses, etc. The experimental insect, *L tristis* (Fig. 1), was collected from various parts of Kerala, India. Insects were collected by hand picking, and after collection they were kept in plastic containers with perforated lids. They were brought to the laboratory for the extraction of defensive gland.

### Extraction of defensive gland secretion

Insects of both sexes were used for milking the defensive secretion. Since the defensive phenolic discharge causes skin burns when extracting gland secretion, latex gloves were used as protection. The beetles were held between the thumb and index finger after displacing and exposing the elytra and terga and then the defensive gland was located. The terminal part of the abdomen was cleaned with cotton soaked in deionized water. A thin needle was used to irritate the beetle, and the defensive glands were extruded out by gently pressing the abdomen. Much care was taken to extrude the gland out and prevent cross-contamination from other substances such as fecal matter. The extract was collected in the Eppendorf tubes (500 µl capacity) containing 300 µl of deionized water. The collected extract was centrifuged at 5000 rpm for 2 min to get rid of any remaining tissue debris. The supernatant was then used for further analysis^4^.

### Synthesis of AgNPs

**Figure 2:**
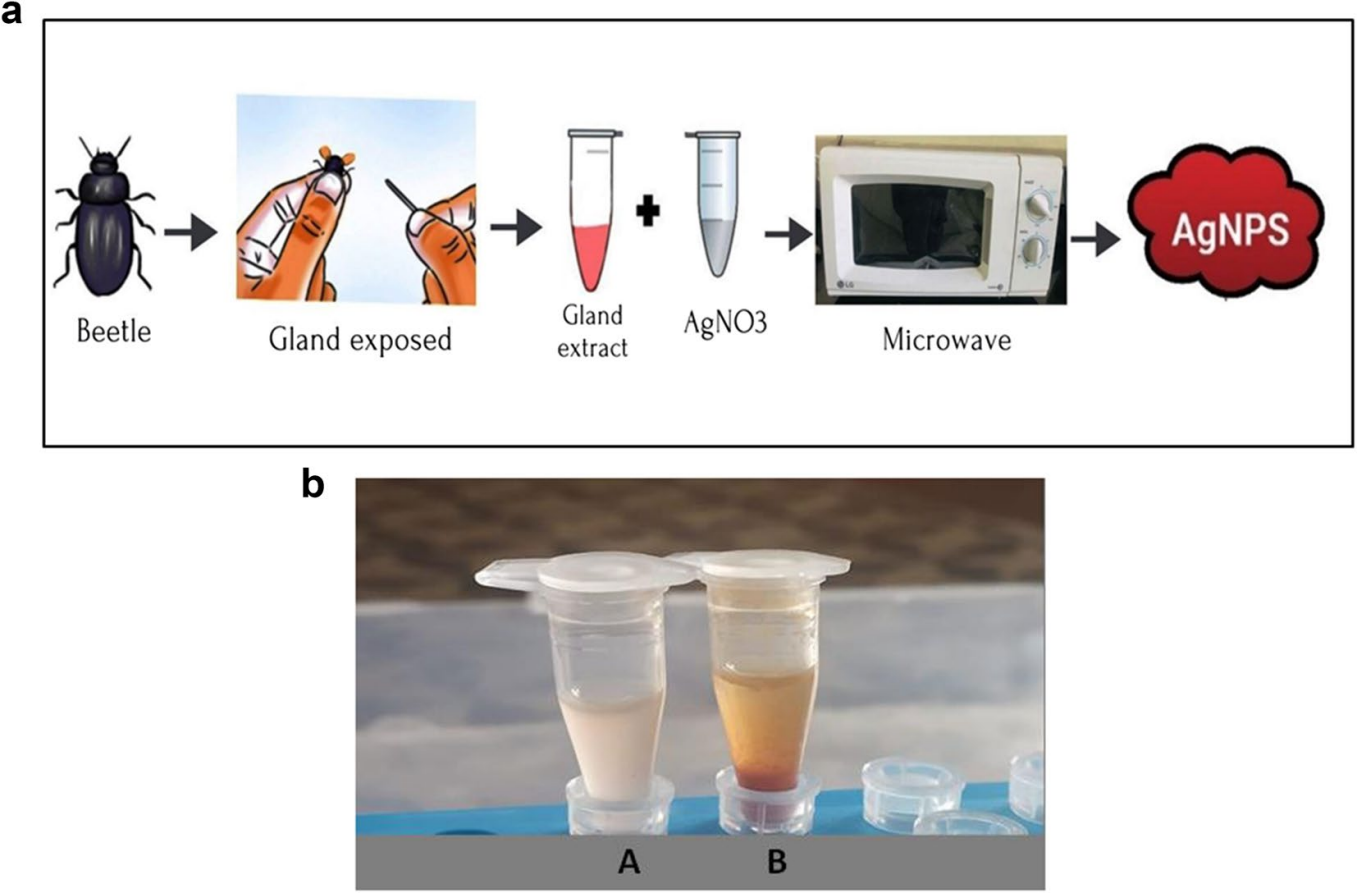
(**a**) Diagrammatic representation of AgNPs synthesis from the defensive gland extracts of *L. tristis*. (**b**) Reaction mixture of AgNO_**3**_ and defensive gland extract before heating (**A**). Reaction mixture after heating for 6 min showing the formation of AgNPs (**B**).

AgNPs were prepared by adding gland extract of 30 beetles in 300 µl of distilled water. AgNO_3_ solution of 0.01 mM (mM) was prepared in deionized water. The reaction mixture was prepared by adding 300 µl of defensive gland extract (30 gland equivalents) and 200 µl AgNO_3_ (0.01 mM). It was heated in a domestic microwave oven (LG-MS-2029 UW) operating at a microwave power level of 350 W for 6 min. A noticeable color change in the solution from pale pink to dark brown after 6 min indicates the synthesis of AgNPs (Fig. 2b). A diagrammatic representation of the synthesis of AgNPs is presented in Fig. 2a. The AgNPs were purified using the dialysis technique for 72 h. The purified AgNPs were stored in room temperature for further characterization and bioassays^4^.

### Characterization of nanoparticles

The formation of nanoparticles was determined by UV–Vis spectroscopy analysis. The absorbance of the solution was measured using a spectrophotometer (PerkinElmer UV/Vis spectrometer).

#### Fourier-transform infrared spectroscopy

FTIR analysis was carried out to investigate the interactions between the functional groups present in the beetle gland extract as a source of reducing and stabilizing agents on the surface of biosynthesized AgNPs. To gain a better understanding of capping and stabilizing molecules, the biosynthesized AgNPs were filtered using a dialysis membrane with a molecular weight cut-off (MWCO) of 3.5 kDa for a period of 72 h. After that, FTIR analysis was performed on three samples: defensive gland extract, AgNPs, and AgNPs after dialysis.The samples were completely dried and ground with KBr pellets and analyzed using an FTIR instrument (PerkinElmer, FTIR spectrometer). The spectra obtained were plotted as transmittance (%) versus wave number (cm^−1^).

#### High-resolution transmission electron microscopy

HR-TEM is a powerful tool used to determine the synthesized AgNPs exact size, morphology and shape. The biologically synthesized AgNPs were analyzed on an HR-TEM instrument (Jeol/JEM 2100).

#### SEM–EDX analysis

SEM–EDX is a common technique used to study morphological and surface characterization and examine metal particle size at the nano- to micro-level scale. Synthesized AgNPs were analyzed on an SEM–EDX instrument (Jeol 6390LA/OXFORD XMX N).

#### Zeta potential analysis

A Zeta potential analysis instrument (Horiba Scientific SZ-100, Japan) was used to analyze the stability of biologically synthesized AgNPs.

#### Analysis of antimicrobial activities

In vitro antibacterial activity of the gland extract of Mupli beetle was studied by agar disc diffusion assay method. To test the antibacterial activity, *S. aureus* and *E. coli*—two prominent Gram-positive and Gram-negative bacteria—were selected. These pathogens cause various diseases in human beings. Mueller Hinton agar (MHA) well diffusion method was employed to investigate the antibacterial activity. Spore suspension of bacteria (10^6^ CFU ml^−1^) was added to a sterile Muller Hinton medium before solidification. It was then poured into sterile Petri dishes (9 cm in diameter) and spread using a cotton swab. From a stock solution of 1 µg/µl, different quantities (5 µl, 10 µl and 15 µl) of biosynthesized AgNPs were pipetted into sterile discs of 6 mm and placed at the center of the Petri dishes from a stock solution of 1 µg/microliter. The antibiotic kanamycin was used as a positive control in the experiment. The Petri dishes were incubated for 16 h at 37 °C. The zone of inhibition was analyzed to estimate the antibacterial effect^4^.

#### Anticancer activity

The cytotoxicity of biologically synthesized AgNPs was analyzed as previously reported^4^. The analysis was carried out in Amala cancer research centre, Thrissur, Kerala, India. A short-term in vitro cytotoxicity assay was carried out using DLA cells.

#### Statistical analysis and data representation

Results obtained from the cytotoxicity assay were represented as percentages of cytotoxicity ± standard deviation, and a Microsoft Excel program was used to plot the results graphically. The concentration of sample required to produce 50% scavenging activity (EC_50_) was analyzed from the graph through linear regression analysis. The results obtained from the DPPH assay were represented graphically by Origin data analysis software.

## Results and discussion

The AgNPs were synthesized by mixing the defensive gland extract of *L. tristis* and silver nitrate (AgNO_3_) solution at 350 W microwave powers. The color changes from light yellow to orange noticed at 6 min (*t* = 6 min) indicate the formation of AgNPs (Fig. 1). This is the first report on the synthesis of a metal nanoparticle using the defensive gland extract of beetle. In a previous research, snail slime extracted from *Helix aspersa* was used to stabilize and reduce AgNO_3_ to AgNPs^32^. Honey made by honey bees was employed as a reducing and capping agent during the fabrication of gold nanoparticles. High-resolution transmission electron microscopy (HR-TEM), X-ray diffraction (XRD), and ultraviolet–visible (UV–Vis) spectra have demonstrated that the biosynthesized spherical AgNPs have a size of around 15 nm^33^. Previous studies have also documented the production of AgNPs from the wing extract of an insect *Mang Mao*, which also exhibited antibacterial and antioxidant properties^34^. We can scale up the production of metal nanoparticles by either extracting the defensive secretion of the beetle in massive quantities when it is available or by artificially synthesizing chemical compounds identified in the extract.

### Characterization of AgNPs

The characterization of AgNPs was carried out using UV spectroscopy, Fourier-transform infrared spectroscopy (FTIR), scanning electron microscopy energy-dispersive X-ray (SEM EDX) and HR-TEM analysis.

### UV analysis

**Figure 3:**
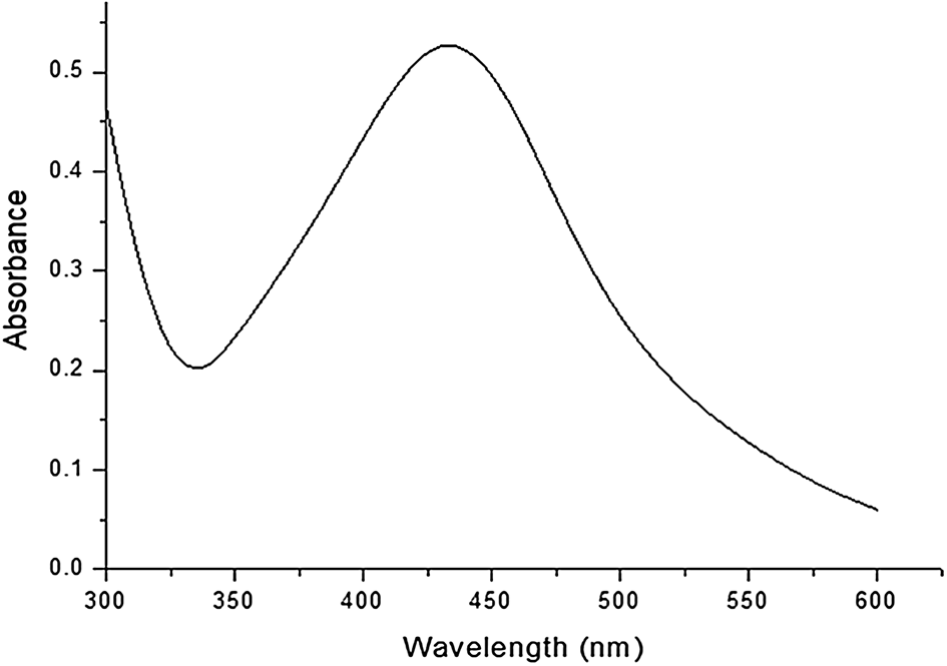
UV–Vis spectrum showing absorbance at 448 nm.

During the green synthesis of AgNPs, a noticeable color change in the solution from pale pink to dark brown indicates the presence of AgNPs (Fig. 2b). The results of the UV–Vis analysis are presented in Fig. 3. As seen in the spectrum, a strong peak at 448 nm indicates the Surface Plasmon Resonance peak that arises from the oscillations of surface electrons of silver metal in an electromagnetic environment. Thus, the formation of AgNPs using the defensive secretion of *L. tristis* is confirmed.

### FTIR analysis

**Figure 4:**
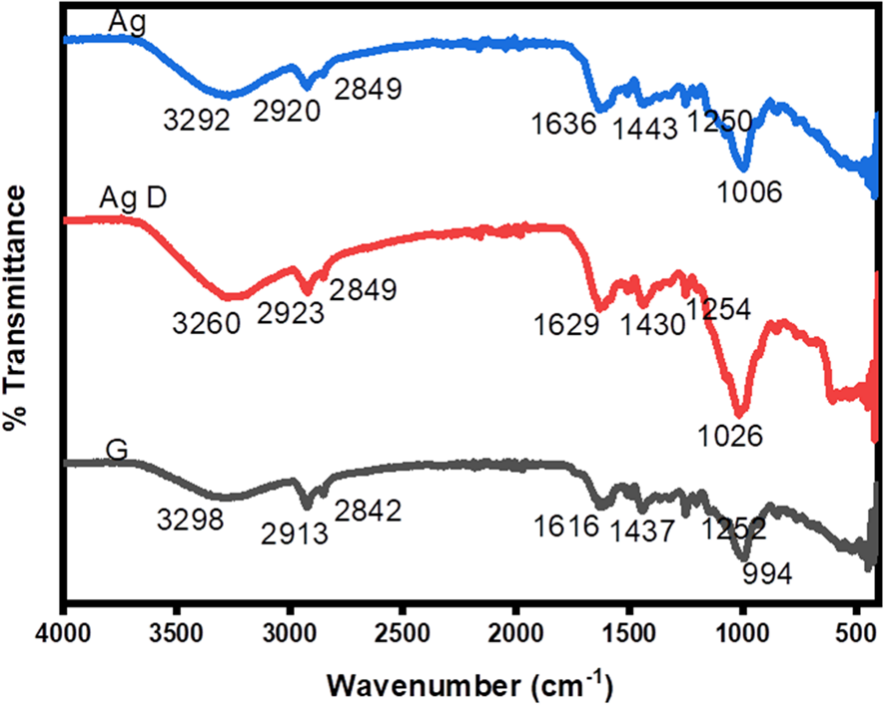
FTIR data of biosynthesized AgNPs after dialysis (Ag), AgNPs before dialysis, and defensive gland extract (G).

FTIR measurements were carried out to identify the presence of different functional groups in the defensive gland extract responsible for the reduction of Ag^+^ and capping/stabilization of AgNPs. The IR spectra of defensive gland extract (G), AgNPs before dialysis (Ag), and AgNPs after dialysis (AgD) were analyzed (Fig. 4). The observed intense bands were compared with the standard values to identify the functional groups (Fig. 4). The IR spectrum of the defensive gland extract of the beetle (represented as G) revealed the presence of hydroxyl groups, with a broad peak at 3298 cm^−1^ and a low-intensity C–H alkyl group at 2913 cm^−1^. This extract contained phenolic compounds, as evidenced by the presence of an absorption band at 1636 cm^−1^ brought on by C=C stretching. A similar pattern of spectral bands were identified in the sample, AgNPs before dialysis (Ag), and AgNPs after dialysis (AgD). The results of IR spectrometry clearly demonstrate that the phenolic compounds, 2,3-dimethyl-1,4-benzoquinone, 1,3-dihydroxy-2-methylbenzene, and 2,5-dimethylhydroquinone present in the defensive gland extract of *L. tristis* are thought to be the key reducing, capping, and stabilizing agents of AgNPs^4,35^. Moreover,the defensive gland also contains pheromones^4^, and we identified C-H stretch of hydrocarbon at 2913 cm^−1^, 2842 cm^−1^, in gland extract (G), 2923 cm^−1^ in AgNPs before dialysis, and 2920 cm^−1^ in AgNPs following dialysis. Since the capping molecules (defensive gland extract) is hydrophilic, we also observed H–O–H scissoring and O–H stretching of water molecules.

### Zeta potential analysis

**Figure 5:**
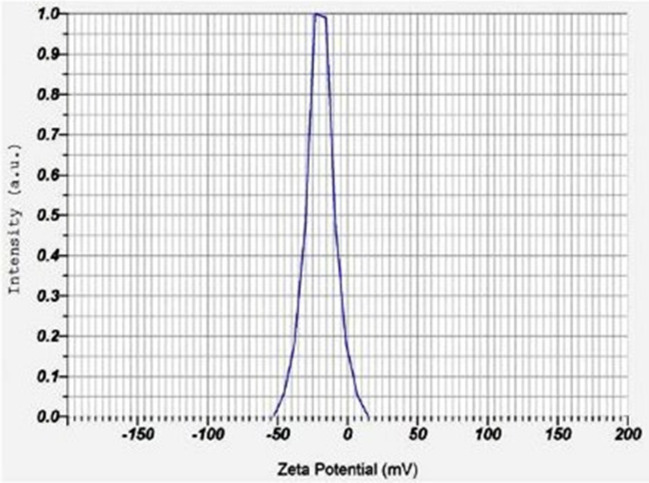
Zeta potential analysis displays the surface charge on AgNPs.

The analysis was carried out to find the available surface charge of AgNPs, which is related to their stability. (Fig. 5). AgNPs synthesized using beetle extract showed a negative zeta potential of − 19.3 mV; greater negative zeta potential values indicate nanoparticles that have enough surface charge to be electrostatically stabilized and resistant to spontaneous aggregation^36^. Almost similar zeta potential values were reported for green synthesized AgNPs utilizing the *Calliandra haematocephala* leaf extract, *Achillea biebersteinii* flowers extract, and aqueous extracts of three *Sideritis* species from Turkey^37–39^.

### HR-TEM analysis

**Figure 6:**
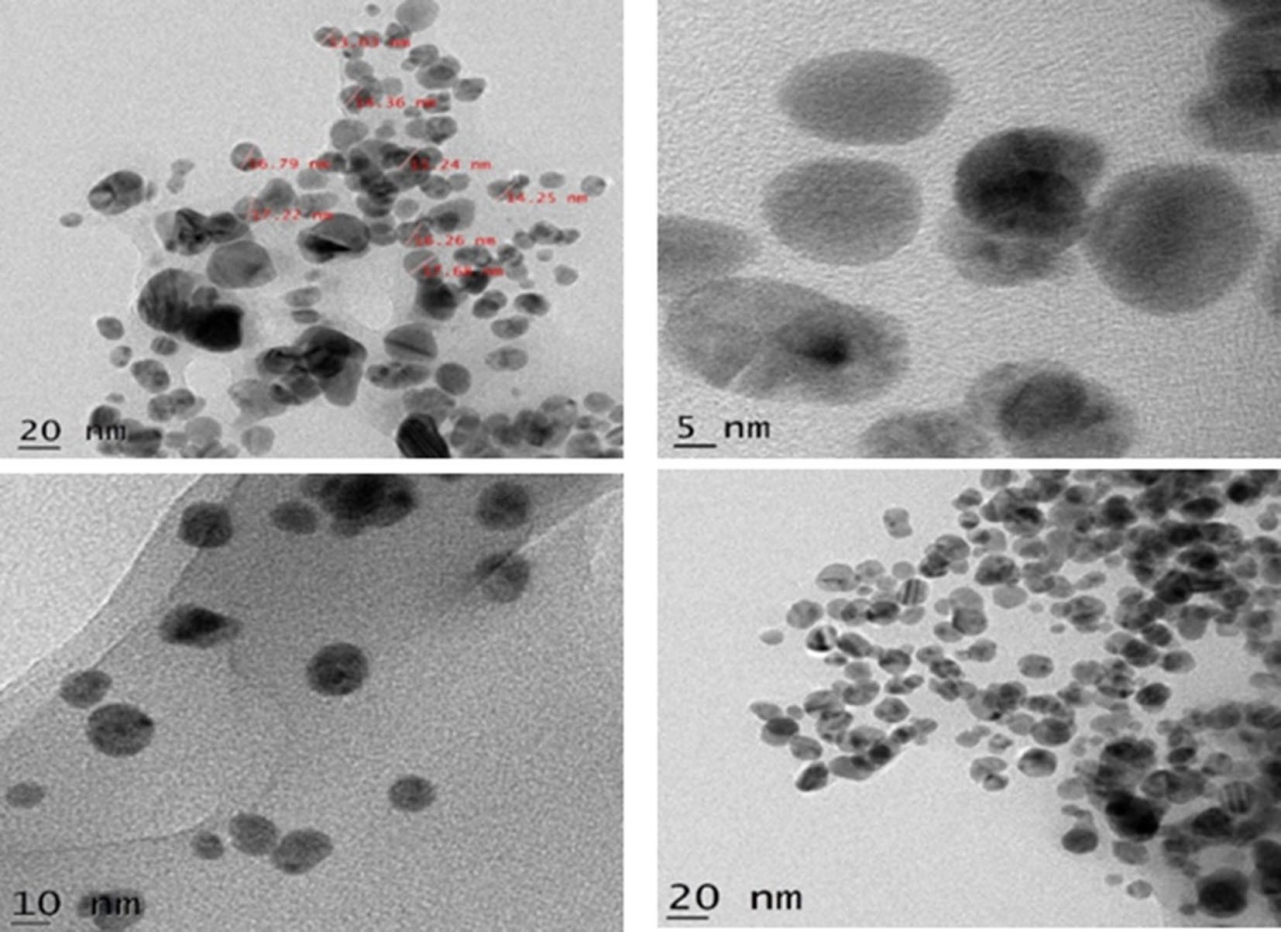
HR-TEM data of different magnifications of AgNPs synthesized from the defensive gland extract of the Mupli beetle.

The exact size, morphology, and shape of synthesized AgNPs were analyzed in detail by HR-TEM. As shown in Fig. 6, the AgNPs are well dispersed and almost round shaped. The average size of the biosynthesized AgNPs ranges from 10 to 20 nm. In the previous study, AgNPs with comparable size and shape were synthesized by the green synthesis method using *Entada rheedii* leaf extract^40^. A snail slime extract-based green synthesis of AgNPs revealed the formation of uniformly round-shaped AgNPs with a minimum and maximum size ranging from 5 to 80 nm^10^.

### SEM–EDX analysis

**Figure 7:**
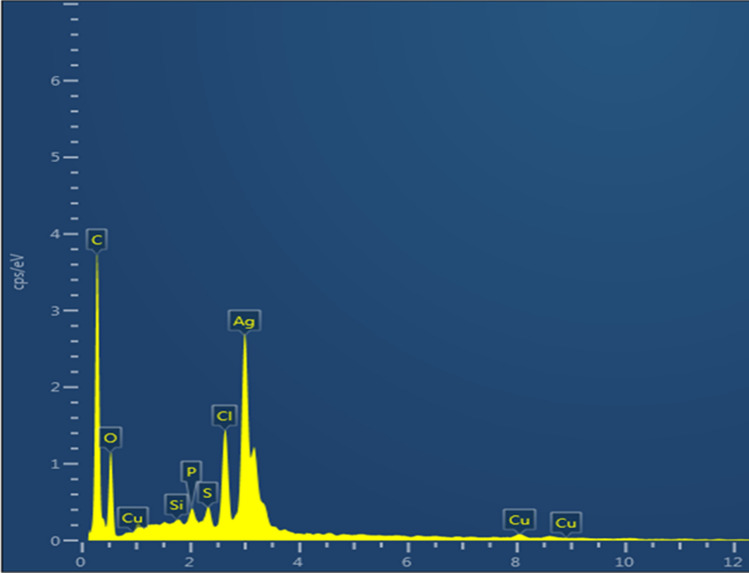
SEM–EDX spectrum of AgNPs elemental mapping results indicates the distribution of elements.

In the SEM–EDX analysis of biologically synthesized AgNPs, strong signals were observed from the silver atoms in the nanoparticles at 3 keV, which is a characteristic peak of metallic silver nanocrystals (Fig. 7). The results were in line with the previous reports on the EDX spectra of green synthesized AgNPs^41^. Other signals were originated from carbon and copper, which is because of the SEM grid.

### Mechanism prediction for AgNP formation

**Figure 8:**
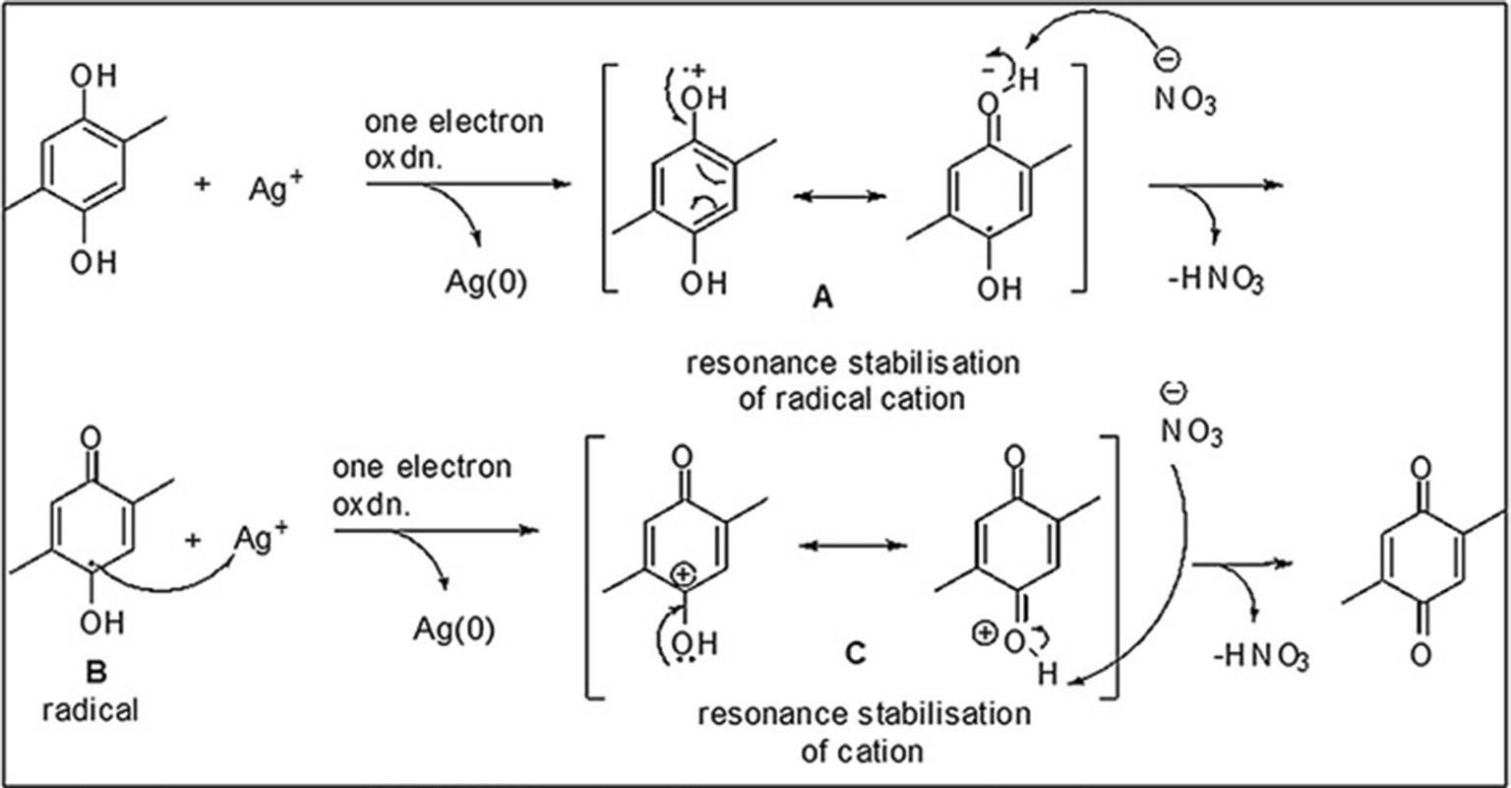
Mechanistic rationalization for the reduction of Ag(I) by 2,5-dimethyl-hydroquinone and 1,3-dihydroxy-2-methylbenzene.

A simplified mechanistic rationale for the reduction of Ag(I) to silver metal by 2,5-dimethylhydroquinone present in the defensive gland extract^4^ is depicted in Fig. 8. It is generally known that hydroquionones are readily oxidized to corresponding *p*-quinones by a number of oxidizing agents, including Ag(I). AgNO_3_ is often used as a reagent for this oxidation. It is reasonable to infer that the initial step in this conversion is the single electron oxidation of the hydroquinone by Ag(I) to afford a transient cation radical **A** and silver metal. The cation radical **A** is stabilized by resonance as indicated in Fig. 8. The removal of a proton from the cation radical **A** then generates a neutral, free radical **B**. The latter is presumably rapidly oxidized by Ag(I) to produce a resonance stabilized carbocation **C**. Subsequent removal of a proton from **C** affords the final products of the oxidation, the dimethyl-*p*-quinone and metallic silver. It may be noted that two moles of Ag(I) are consumed during the oxidation of one mole of hydroquinone.

### DPPH scavenging activity

**Figure 9:**
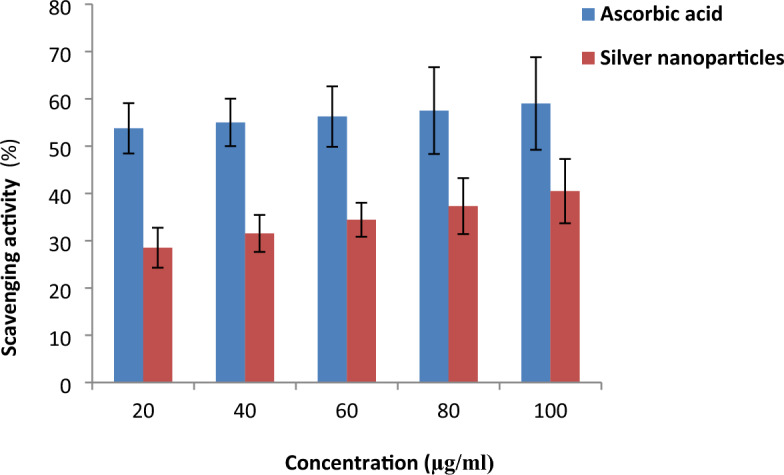
DPPH activity of various concentrations of AgNPs.

DPPH is a purple-colored free radical that turns yellow when given a proton by an antioxidant^42^. In the present investigation, the biosynthesized AgNPs in an aqueous suspension demonstrated dose-dependent DPPH radical scavenging action with an IC_50_ value of 162.64 µl (Fig. 9). Phenolic compounds were identified in the *L. tristis* defense gland extract by HR-GC MS analysis^4^. Phenolic compounds have antioxidant property due to their abilities as reducing agents, radical scavengers, and hydrogen donors. They are effective antioxidants as they can donate hydrogen atoms from their aromatic hydroxyl groups to free radicals and have a resonance effect in their aromatic rings^43^. Metal nanoparticles synthesized from plant leaf extracts showed good free radical scavenging activity as they contain phenolic compounds^42,44,45^.

### Anticancer activity of AgNPs

**Figure 10:**
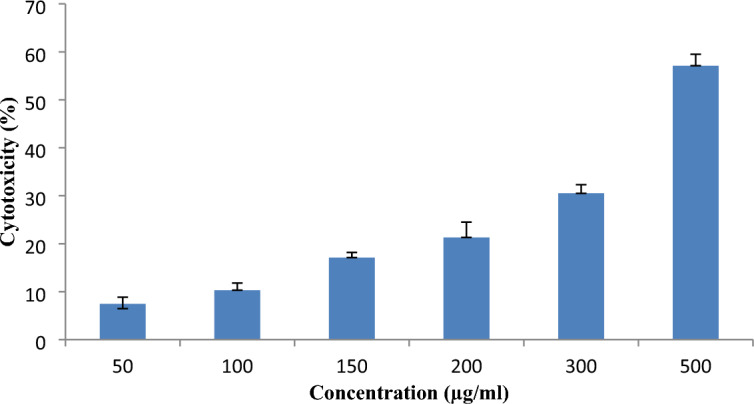
The effect of AgNPs on the cytotoxic activity of DLA cells was estimated by trypan blue assay.

Figure 10 shows the in vitro cytotoxic property of AgNPs at various doses (50, 100, 150, 200, 300, and 500 µg ml^−1^) on Dalton’s lymphoma ascites (DLA) cells using the trypan blue assay. The results demonstrated that cytotoxicity increased from lower to higher concentrations in a dose-dependent manner. Similar effect was noticed in a terrestrial snail; mucus-mediated green synthesized AgNPs were found to have a dose-dependent cytotoxic effect on HeLa (cervical carcinoma) cells^46^. AgNPs promote the production of reactive oxygen species, which induces oxidative damage to cellular components such as DNA, proteins, and lipids and ultimately leads to cell death^47,48^. Numerous biomolecules, in particular, polyphenolic compounds from the defensive gland extracts of *L. tristis* adsorbed onto the surface of AgNPs, may be responsible for the anticancer activity even at lower concentrations (50 µg ml^−1^).

### Antibacterial activity of AgNPs

**Figure 11:**
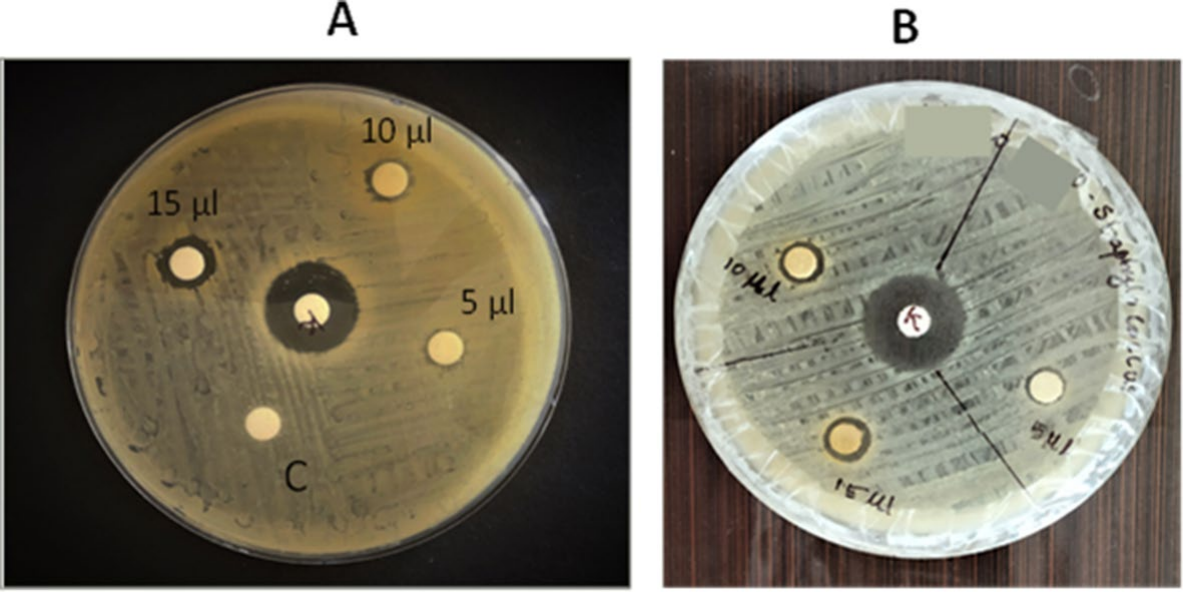
Antimicrobial activity of different concentrations (5 µl, 10 µl, and 15 µl) of AgNPs on *E. coli* (**A**) and *S. aureus* (**B**).

Antibacterial activity of AgNPs synthesized from Mupli beetle gland extract was studied by agar disc diffusion assay method. The findings of the experiment presented in Fig. 11A,B imply that the biosynthesized AgNPs exhibit antibacterial properties against the bacteria *S. aureus* and *E. coli*. The antibacterial activity of AgNPs is attributed to three different antibacterial mechanisms, which include: (a) the uptake of silver ions by bacteria, which prevents ATP synthesis and DNA replication; (b) AgNPs’ ability to cause oxidative stress; and (c) AgNPs induce direct damage to the bacterial cell membranes, which results in bacterial cell lysis^41,49–51^.

## Conclusion

In the current study, microwave-assisted AgNP synthesis employing the defensive gland extract of the Mupli beetle, *L. tristis*, addresses two key challenges faced by the field of nanoparticle synthesis. The study was conducted without killing the experimental insect. The data show that the defensive gland extract containing phenolic compounds have the ability to reduce silver ions into AgNPs; moreover, they act as a good capping and stabilizing agent. A possible mechanism for the reduction of AgNO_3_ into AgNPs is put forth using the computational software QCHEM, and it suggests the reduction of either AgOH or AgNO_3_. The synthesized AgNPs were characterized by UV–Vis spectroscopy, FTIR, SEM–EDX, TEM, and zeta potential analysis. The structure analysis confirmed that AgNPs are well dispersed and almost round shaped. The average size of nanoparticles ranges from 10 to 20 nm. The AgNPs synthesized have the antibacterial property against both *E. coli* and *S. aureus* and they also exhibited antioxidant activity in DPPH assay. The AgNPs showed anticancer activity in a cytotoxicity experiment against the DLA cell line. The advantages of employing Mupli beetle defense extract for the production of AgNPs were cost-effectiveness, energy efficiency, environmental friendliness, and human health-friendliness. All things considered, we may conclude that *L. tristis* defensive extract is critical for the production of bioactive AgNPs as reducing and stabilizing agents. Future biological and environmental applications of this eco-friendly technique are possible.

## Data Availability

Data that support the findings of this study are available from the corresponding author upon reasonable request.
